# Factor structure, internal consistency, and measurement invariance of the Eating Pathology Symptoms Inventory (EPSI) in a national U.S. sample of cisgender gay men and lesbian women

**DOI:** 10.1186/s40337-025-01277-z

**Published:** 2025-05-14

**Authors:** Jason M. Nagata, Christopher D. Otmar, Angela E. Kim, Emilio J. Compte, Jason M. Lavender, Tiffany A. Brown, Kelsie T. Forbush, Annesa Flentje, Micah E. Lubensky, Mitchell R. Lunn, Juno Obedin-Maliver

**Affiliations:** 1 Department of Pediatrics, University of California, San Francisco, 550 16th Street, 4th Floor, Box 0503, San Francisco, San Francisco, CA 94143 USA; 2 Eating Behavior Research Center, School of Psychology, Universidad Adolfo Ibáñez, Santiago, Chile; 3 Research Department, Comenzar de Nuevo Treatment Center, Av. Humberto Lobo 1001a, Del Valle, San Pedro Garza García, 66220 N.L Mexico; 4 Military Cardiovascular Outcomes Research Program (MiCOR), Department of Medicine, Uniformed Services University of the Health Sciences, 4301 Jones Bridge Rd, Bethesda, MD 20814 USA; 5 The Metis Foundation, 84 NE Interstate 410 Loop # 325, San Antonio, TX 78216 USA; 6 Department of Psychological Sciences, Auburn University, Thach Hall, Suite 226, Auburn, AL 36849 USA; 7 Department of Clinical Child Psychology, University of Kansas, Lawrence, KS USA; 8 The PRIDE Study/PRIDEnet, Stanford University School of Medicine, 300 Pasteur Drive, Grant Building S102, Stanford, MC, CA 5110, 94305 USA; 9 Department of Community Health Systems, University of California, San Francisco, 490 Illinois Street, Floor 9, Box 0608, San Francisco, CA 94143 USA; 10 Alliance Health Project, Department of Psychiatry and Behavioral Sciences, University of California, San Francisco, 675 18th St, San Francisco, CA 94107 USA; 11 Division of Nephrology, Department of Medicine, Stanford University School of Medicine, 3180 Porter Dr, Palo Alto, CA 94304 USA; 12 Department of Epidemiology and Population Health, Stanford University School of Medicine, 1701 Page Mill Rd, Palo Alto, CA 94304 USA; 13 Department of Obstetrics and Gynecology, Stanford University School of Medicine, 453 Quarry Road, Palo Alto, CA 94304 USA

**Keywords:** Eating disorders, Sexual minority health, Measurement invariance, Scale validation, Factor analysis, LGBTQIA+

## Abstract

**Background:**

The Eating Pathology Symptoms Inventory (EPSI) is a questionnaire that assesses the severity of eating-disorder symptoms. This study aimed to examine the factor structure and measurement invariance of the EPSI in a large national U.S. sample of cisgender gay men and lesbian women.

**Methods:**

The sample consisted of 1,498 cisgender sexual minority adults, including cisgender gay men (*n* = 925) and cisgender lesbian women (*n* = 573), who completed online self-report surveys. Using a split-half approach, exploratory factor analysis (EFA) was conducted in the first subset of each sample to identify underlying factor structures, followed by confirmatory factor analysis (CFA) to confirm model fit in the second subset of each sample. Multi-group confirmatory factor analysis (MG-CFA) was used to assess measurement invariance across the two sexual minority groups.

**Results:**

The EPSI eight-factor structure was supported across both cisgender sexual minority groups with strong model fit: cisgender gay men (CFI = 0.96, RMSEA = 0.04, SRMR = 0.06) and cisgender lesbian women (CFI = 0.94, RMSEA = 0.05, SRMR = 0.07). Measurement invariance analyses indicated that the EPSI was invariant across groups. Internal consistency, assessed using McDonald’s omega, was acceptable for all scales (ωs = 0.75 to 0.95).

**Conclusions:**

This study provides support for the utility of the EPSI in cisgender gay men and lesbian women populations, including measurement invariance that allows for meaningful comparisons across groups. Specifically, the EPSI performs reliably and consistently as a measure of eating pathology across adult cisgender gay men and cisgender lesbian women.

**Supplementary Information:**

The online version contains supplementary material available at 10.1186/s40337-025-01277-z.

## Abstract (es)

Cisgender (those whose sex assigned at birth matches their gender identity) sexual minority adults (those with a sexual identity other than exclusively heterosexual) in the U.S. are at higher risk for disordered eating than their heterosexual counterparts. The Eating Pathology Symptoms Inventory (EPSI) is a questionnaire measuring disordered eating, but its utility among cisgender gay men and cisgender lesbian women is unknown. EPSI scores demonstrated evidence of reliability and validity in this U.S. sample of adult cisgender gay men and lesbian women.

## Background

Sexual minority adults are at an elevated risk of experiencing eating pathology [1, 2] and are two to four times more likely than their heterosexual peers to receive a clinical diagnosis of an eating disorder [3]. These disparities are evident across cisgender gay men and cisgender lesbian women [4–6]. Eating disorder symptoms often co-occur with other mental-health concerns, including poorer psychosocial functioning [7–9] and greater risk of substance use [10–12] and suicidal ideation [13]. Sexual minority stigma-based stress can amplify these pathways [14, 15]. Indeed, cisgender gay men and cisgender lesbian women face unique stressors in their daily lives [16], such as internalized stigma and interpersonal discrimination, that can contribute to eating-disorder symptoms and negatively affect overall quality of life and psychosocial health. Significant structural barriers impede cisgender gay men and cisgender lesbian women from accessing the healthcare needed to address eating disorder-related challenges [17]. Although there is growing recognition of the escalating rates of eating disorders within sexual minority communities [1], limited research has: (1) examined the factor structure of newer eating disorder measures that are used to assess symptom severity and (2) assessed whether these measures operate equivalently across cisgender gay men and cisgender lesbian women. This is especially critical for measures that employ multiple items to comprehensively capture the varied symptoms associated with eating pathology [18]. Therefore, the primary aim of this manuscript was to examine the factor structure, internal consistency, and measurement invariance of the Eating Pathology Symptoms Inventory (EPSI) to ensure that it functions similarly across cisgender gay men and cisgender lesbian women groups, allowing for equitable and accurate interpretation of symptom severity.

Research supports pronounced divergences in sex-based manifestations of eating pathology, although this is likely due at least in part to differences in sociocultural influence and gender norms [19, 20]. For example, cisgender men may engage in muscle-enhancing behaviors like excessive weightlifting and the use of muscle-enhancing supplements [21], whereas predominantly restrictive eating patterns tend to be more prevalent in cisgender women [22]. Some symptoms are consistent across sexual identity and appear to be driven by gender-related cultural norms. Objectification Theory [23] suggests that sociocultural norms surrounding physical appearance and beauty ideals heighten body surveillance, particularly among vulnerable groups [21, 24]. This theory may help explain why patterns of eating disorders vary by biological sex and are further influenced by culturally constructed gender norms. Wiseman and Moradi (2010) found that, although (presumably cisgender) sexual minority men were more likely than (presumably cisgender) heterosexual men to exhibit symptoms related to weight concerns, they were equally as likely to show symptoms related to muscle-building [21]. Additionally, cisgender sexual minority women had higher levels of binge-eating behaviors than cisgender heterosexual women, but both groups showed similar levels of body dissatisfaction and internalization of gender-specific sociocultural beauty standards [22].

Given elevated risk among cisgender gay men and cisgender lesbian women, understanding the nature of eating pathology and how symptoms may differ between groups is important. However, it is first necessary to ensure that measures used to assess eating pathology are psychometrically sound in these populations. The psychometric properties of many eating disorder measures have been understudied, particularly among LGBTQIA + communities. To facilitate future research, there is a need to evaluate existing measures of eating disorder symptoms to understand their psychometric performance in samples with diverse sexual orientations and gender identities.

The EPSI, published in 2013, is a 45-item questionnaire [25] that assesses multiple dimensions of eating pathology, including body dissatisfaction, binge eating, cognitive restraint, purging, restricting, excessive exercise, negative attitudes toward obesity, and muscle building [25]. The EPSI has been validated across various age groups [26–29] and translated into multiple languages [26–30]. Recently, Perko et al. (2021) tested the factor structure of the EPSI in a large sample of (presumably cisgender) gay and bisexual men and found support for the original eight-factor structure [31].

Building on the existing literature [32], the aim of this study was to examine the factor structure, internal consistency, and measurement invariance of the EPSI in national U.S. samples of cisgender gay men and cisgender lesbian women [25, 30, 31].

## Methods

### Procedure

We utilized data from The PRIDE Study (Population Research in Identity and Disparities for Equality), a national, longitudinal, community-engaged, cohort study examining sexual and gender minority (SGM) adult health and well-being. Eligibility criteria included being at least 18 years old, self-identifying as a member of the SGM community, residing within the U.S. or its territories, and having the ability to read and understand English. Data collection was facilitated through a secure, cloud-based platform accessible via any internet-connected device. Recruitment strategies involved outreach through PRIDEnet, social media campaigns, community blogs, events, and word-of-mouth referrals. Detailed descriptions of the study design and recruitment methodologies, as well as community engagement model, are available in Lunn et al. (2019) [33] and Obedin-Maliver et al. (2024) [34], respectively. Between July 2023 and January 2024, all participants enrolled in The PRIDE Study were invited to complete the “Eating and Body Image 2023,” which was developed to assess a wide range of eating disorder topics. Respondents were entered into a lottery to win one of 50 gift cards valued at $40 each with an approximate chance of 1 in 95 to win. The Institutional Review Boards of Stanford University School of Medicine (#63400), the University of California, San Francisco, and WIRB-Copernicus Group (WCG) approved this study with oversight from The PRIDE Study’s Research and Participant Advisory Committees. Participants provided informed consent prior to engaging in study-related procedures.

### Participants

A total of 4,729 individuals completed the ‘Eating and Body Image 2023’ survey, which comprised the EPSI and a number of other eating-disorder measures to comprehensively assess disordered eating behaviors and attitudes, such as the SCOFF questionnaire [35], the Eating Disorder Diagnostic Scale [36], and the Muscularity-Oriented Eating Test [37]. The current study focused specifically on the EPSI among cisgender gay men (*n* = 925) and cisgender lesbian women (*n* = 573); therefore, only participants who selected “gay/lesbian” for sexual orientation (*n* = 1,896, 41.1%), and “cisgender man” (*n* = 1,122, 24.4%) or “cisgender woman” (*n* = 1,379, 30.0%) for gender identity were included in the present analysis. Sexual orientation was determined using a single self-report question: “If you had to choose only one of the following terms, which best describes your current sexual orientation?” and gender identity was determined using a single self-report question: “If you had to choose only one of the following terms, which best describes your current gender identity?”

Among the final sample of participants (*N* = 1,498), the mean age was 50.78 years (*SD* = 15.2, range = 18–96). The majority of participants identified as White (*n* = 1,251, 84.8%), followed by Hispanic, Latino, or Spanish (*n* = 75, 3.6%), Black or African American (*n* = 55, 2.6%), and Asian (*n* = 48, 2.3%) and American Indian or Alaska Native (*n* = 45, 2.1%). Participants also identified as Middle Eastern or North African (*n* = 11, 0.5%), and Native Hawaiian or Pacific Islander (*n* = 2, 0.1%). Other or unknown racial/ethnic categories accounted for 0.5% (*n* = 11) of the sample. Participants could select multiple racial/ethnic identities, and 9.3% of participants (*n* = 140) reported more than one ethnoracial identity (cisgender gay men, *n* = 89, 9.6%; cisgender lesbian women, *n* = 51, 8.9%). Most participants had completed a college degree or higher (*n* = 1,192, 79.7%), whereas a smaller portion of participants had less than a college degree (*n* = 305, 20.3%).

### Measures

The EPSI is a 45-item multidimensional self-report assessment designed to assess a wide range of eating-related behaviors and attitudes experienced within the past four weeks including Body Dissatisfaction (7 items), Binge Eating (8 items), Cognitive Restraint (3 items), Purging (6 items), Restricting (6 items), Excessive Exercise (5 items), Muscle Building (5 items), and Negative Attitudes Toward Obesity (5 items). All items are rated on a 5-point Likert-type scale ranging from 0 (never) to 4 (very often) [25]. Scale scores are calculated by summing the items’ scores within each factor; higher scores indicate a greater frequency of symptoms. Importantly, the EPSI is not intended to diagnose eating disorders as it lacks clinical cutoff scores [38]. Previous psychometric studies on the EPSI indicated strong internal consistency for most scales with Cronbach’s alpha typically ranging from 0.78 to 0.95 [25, 30]. The Muscle Building scale has shown comparatively lower reliability (α = 0.54–0.84), especially among women. Convergent validity has been evidenced by moderate-to-high correlations (*r* =.51–0.74) with the Eating Disorder Examination Questionnaire (EDE-Q) and smaller but significant correlations (*r* =.22–0.58) with the Eating Attitudes Test (EAT-26) [25, 28]. Discriminant validity is supported by lower correlations (*r* =.17–0.56) with measures of anxiety and depression [26]. Internal consistency of the EPSI scale scores was assessed using McDonald’s Omega (ω) in the current investigation.

### Data analyses

Data were analyzed using several statistical packages in R (version 4.4.0). Descriptive statistics and internal consistency of the EPSI scale scores was computed with the ‘psych’ package (version 2.4.6.26) [39]. Multivariate normality was assessed with Mardia’s test using the ‘MVN’ package (version 5.9) [40], and factor analyses and measurement invariance testing were performed using the ‘lavaan’ package (version 0.6–19) [41]. For each group, participants were randomly divided into split-half subsamples to conduct EFA and CFA. To manage non-normal data distributions, we used weighted least squares mean and variance adjusted (WLSMV) estimation for each of the factor analyses. EFAs used oblique Oblimin rotation to allow for correlated factors because previous studies on the EPSI scale scores have demonstrated that eating-related behaviors and attitudes are interrelated. We used parallel analysis to inform the number of factors to retain in the EFAs conducted with data from the first split-half subsamples. Parallel analysis compares observed eigenvalues to eigenvalues derived from randomly generated datasets, and factors for which the observed values are greater than the randomly generated values are retained. CFAs were then conducted within the second split-half subsamples of each group. Model fit was determined using the following criteria: Root Mean Square Error of Approximation (RMSEA) less than 0.06 with 90% confidence intervals, Standardized Root Mean Square Residual (SRMR) less than 0.08, and Comparative Fit Index (CFI) and Tucker-Lewis Index (TLI) greater than 0.90 [42]. All items were allowed to load freely onto their respective factors with one item per factor fixed to one. This establishes the scale of the latent variable and helps with interpretation of factor loadings.

Following the single-group CFAs, we conducted a multi-group confirmatory factor analysis (MG-CFA) [43], which determined if meaningful comparisons could be made across populations [18, 44]. We performed a series of hierarchical measurement invariance tests across groups: configural, metric, scalar, and residual invariance [32, 45]. Configural invariance indicated that the scale shares a similar factor structure across groups, meaning the construct was conceptualized similarly. Metric invariance added the equivalence of factor loadings across groups, suggesting that the items contribute similarly to the latent variable in each group. Scalar invariance means the equivalence of intercepts. In other words, the observed scores were the same across groups at the same levels of the latent variable. Establishing at least scalar invariance is considered psychometrically acceptable because it allows any observed differences to be directly attributed to differences in the latent construct [18]. The same criteria apply to strict invariance with the additional condition that residual variances are equal across groups.

We applied specific thresholds for each level of invariance testing, following guidelines from Chen (2007) [46]. Metric invariance holds when the change in CFI (ΔCFI) is less than 0.010, the change in RMSEA (ΔRMSEA) is below 0.015, and the change in SRMR (ΔSRMR) is under 0.030. Scalar invariance requires ΔCFI to stay below 0.010, ΔRMSEA below 0.015, and ΔSRMR under 0.010. Strict invariance uses the same criteria, with the additional condition that residual variances are equal across groups.

## Results

### Scale reliability and correlations among cisgender gay men and cisgender lesbian women

The EPSI scale scores demonstrated strong reliability with ω values ranging from 0.75 to 0.95 (see Table 1), however, given concerns that high internal consistency may indicate item redundancy [47], we examined average inter-item correlations (AIC) as an additional check [48]. The AIC values were 0.222 for cisgender gay men and 0.178 for cisgender lesbian women, both well below the 0.50 threshold for redundancy, indicating that the high internal consistency reflects coherence rather than excessive item similarity. Zero-order correlations among the scales are presented in Table 2. The correlations in Table 2 were based on traditionally scored scales rather than latent variable correlations and captured the direct relationships between scales without accounting for measurement error. Factor correlations from the CFA models, which are typically higher due to their basis in latent constructs, are provided in Supplemental Table 1. While the factor correlations suggested interrelationships among the EPSI scales, they also suggested the distinctiveness of the scales.

**Table 1:** Descriptive statistics and reliability for split-half and full samples across two groups of cisgender gay men (*n* = 925) and cisgender lesbian women (*n* = 573)

	Split-Half Sample 1				Split-Half Sample 2				Full Sample		
	Cisgender Gay Men										
	M	SD	ω		M	SD	ω		M	SD	ω
Body Dissatisfaction	11.1	6.4	0.92		11.2	6.4	0.93		11.1	6.4	0.93
Binge Eating	8.2	6.3	0.93		8.4	6.4	0.94		8.3	6.4	0.93
Cognitive Restraint	5.1	2.9	0.74		5.2	2.7	0.75		5.1	2.8	0.75
Purging	0.7	2.4	0.87		0.9	2.3	0.87		0.8	2.3	0.87
Restricting	4.4	4.4	0.86		4.4	4.5	0.87		4.4	4.4	0.87
Excessive Exercise	4.7	4.6	0.89		4.8	4.6	0.90		4.8	4.6	0.89
Negative Attitudes	6.6	4.8	0.92		6.7	4.8	0.91		6.6	4.8	0.92
Muscle Building	4.3	3.8	0.86		4.2	3.9	0.87		4.3	3.9	0.86

	Cisgender Lesbian Women										
	M	SD	ω		M	SD	ω		M	SD	ω
Body Dissatisfaction	13.6	7.2	0.95		13.6	7.3	0.94		13.6	7.3	0.95
Binge Eating	8.1	6.3	0.91		8.2	6.3	0.91		8.8	6.3	0.91
Cognitive Restraint	4.9	2.9	0.79		4.8	2.10	0.79		4.8	2.9	0.79
Purging	0.7	2.2	0.85		0.7	2.1	0.84		0.7	2.1	0.84
Restricting	4.7	4.7	0.89		4.7	4.5	0.89		4.7	4.6	0.89
Excessive Exercise	3.9	4.3	0.87		3.9	4.2	0.88		3.9	4.3	0.88
Negative Attitudes	5.2	4.3	0.91		5.1	4.3	0.92		5.1	4.3	0.92
Muscle Building	2.2	2.4	0.80		2.2	2.5	0.79		2.2	2.5	0.79

Note: M = Mean, SD = Standard Deviation, ω = McDonald’s Omega

Split-Half Samples represent two randomly divided halves of the full sample for EFA/CFA

**Table 2:** Correlations among the eight eating pathology symptoms inventory (EPSI) scales for cisgender gay men and cisgender lesbian women

		1	2	3	4	5	6	7	8
1	Body Dissatisfaction	—	0.57***	0.30***	0.36***	0.25***	0.07	0.43***	0.15**
2	Binge Eating	0.59***	—	0.12**	0.37***	0.14**	0.07	0.39***	0.17***
3	Cognitive Restraint	0.37***	0.22***	—	0.18***	0.16**	0.45***	0.32***	0.26***
4	Purging	0.32***	0.37***	0.18***	—	0.25***	0.11**	0.23***	0.18***
5	Restricting	0.29***	0.19***	0.16***	0.32***	—	0.05	0.17***	0.19***
6	Excessive Exercise	0.15***	0.18***	0.45***	0.22***	0.15***	—	0.15**	0.36***
7	Negative Attitudes Toward Obesity	0.43***	0.40***	0.32***	0.23***	0.18***	0.29***	—	0.12**
8	Muscle Building	0.30***	0.26***	0.24***	0.28***	0.19***	0.43***	0.23***	—

Note. ***p <.01. Lower correlations are cisgender gay men and upper correlations are cisgender lesbian women. Correlations are based on scored scales and not latent scores

### Exploratory factor analysis among cisgender gay men

In the first split-half subsample of cisgender gay men (*n* = 463), the Kaiser-Meyer-Olkin (KMO) index was 0.88. The KMO values for individual items ranged from 0.76 (Item 7) to 0.95 (Item 43) and Bartlett’s test of sphericity was significant (χ^2^(990) = 11130.42, *p* <.001), which indicates that the data were suitable for EFA. The mean item communality suggested that, on average, 54% of the variance in the observed variables was accounted for by the extracted factors. Parallel analysis supported an eight-factor solution, as the first eight eigenvalues from the observed data were higher than those from the randomly generated data, consistent with the recommended threshold for factor retention. The unrotated 8-factor solution accounted for 53.9% of the total variance explained. After applying oblique rotation, the eight-factor solution accounted for 14.69%, 11.40%, 9.43%, 8.36%, 11.44%, 8.67%, 8.71%, and 9.14% of the total variance, respectively. These values were derived by squaring the loadings from the structure matrix, summing across items for each factor, and dividing by the total number of items. Factor loadings from the pattern matrix, which represent the unique contribution of each factor to an item while accounting for inter-factor correlations, ranged between 0.27 and 0.86 across the eight factors (Table 3 and Supplemental Table 2). Six of the 45 items exhibited cross-loadings onto other factors, which is to be expected with a multidimensional scale utilizing this many items and factors. Because factors were allowed to correlate, the sum of the variance explained across factors exceeds the total variance.

**Table 3:** Exploratory factor analysis loading (pattern matrix) in first split-half of cisgender gay men and cisgender lesbian women

Item		Cisgender Gay Men (*n* = 463)									Cisgender Lesbian Women (*n* = 287)							
		Factor Loadings									Factor Loadings							
		1	2	3	4	5	6	7	8		1	2	3	4	5	6	7	8
*Binge Eating*																		
**3**		0.61									0.59							
**9**		0.52									0.57							
**19**		0.80									0.78							
**28**		0.66									0.59							
**37**		0.51									0.57							
**39**		0.79									0.81							
**44**		0.71									**0.67**							**0.48**
**45**		0.74									0.75							
*Body Dissatisfaction*																		
**1**			**0.68**						**0.28**			0.84						
**12**			0.44									0.54						
**18**			0.74									0.75						
**23**			0.63									0.54						
**24**			0.62									0.77						
**25**			0.73									0.83						
**34**			0.62									0.78						
*Cognitive Restraint*																		
**2**				0.48										0.56				
**21**				**0.75**					**0.36**					**0.60**				**0.46**
**40**				0.48										0.86				
*Excessive Exercise*																		
**5**					0.61										0.61			
**8**					0.72										0.84			
**22**					0.28										0.73			
**31**					0.67										0.73			
**41**					0.61										0.65			
*Muscle Building*																		
**7**						0.75									0.65			
**15**						0.66									0.37			
**29**						0.86									0.84			
**32**			**0.38**			**0.41**									0.31			
**35**						0.54									0.51			
*Negative Attitudes*																		
**14**							0.76									0.69		
**20**					**0.41**		**0.79**									0.77		
**26**							0.74									0.75		
**30**							0.65									0.68		
**38**							0.86									0.83		
*Purging*																		
**11**								0.77									0.57	
**13**								0.79									0.57	
**16**								0.27									0.28	
**17**								0.53									0.48	
**27**		**0.28**						**0.29**									0.26	
**42**																	0.72	
*Restricting*																		
**4**									0.71									0.76
**6**									0.73									0.63
**10**									0.64									0.72
**33**									0.83									0.74
**36**									0.62									0.63
**43**								**0.41**	**0.74**									0.48

Note: For ease of interpretation, only factor loadings > 0.25 are displayed. All cross-loadings are bolded. Full matrices are available in supplemental material

### Confirmatory factor analysis among cisgender gay men

**Fig. 1:**
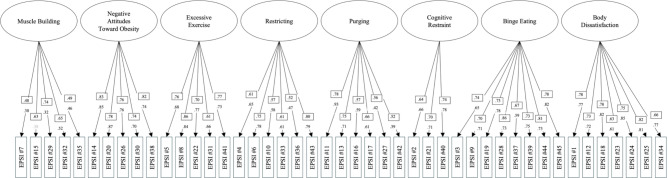
Standardized factor loadings from the confirmatory factor analysis for cisgender gay men and cisgender lesbian women. Note: Factor loadings inside the squares represent values for cisgender gay men factor; values for cisgender lesbian women are not enclosed in squares. Gray indicates factor loading of *p* >.05. Correlations among factors are not depicted for visual clarity

Prior to running the CFA, we assessed multivariate normality using Mardia’s test, which indicated significant deviations from normality (*p* <.001). An eight-factor CFA model was conducted using the second split-half subsample of cisgender gay men (*n* = 462). The initial model demonstrated a good fit to the data (χ^2^(917) = 1545.34, CFI = 0.96, TLI = 0.96, and RMSEA = 0.04 (90% CI = 0.04, 0.05). The SRMR was 0.06, consistent with an acceptable level of fit. These results suggest that the eight-factor model provided a good fit to the data after accounting for the robust correction. Each item significantly contributed to its corresponding latent factor as all factor loadings were statistically significant (*p* <.001) and greater than 0.30. Standardized factor loadings from the CFA are presented in Fig. 1.

### Exploratory factor analysis among cisgender lesbian women

In the first split-half subsample of cisgender lesbian women (*n* = 287), the KMO index was 0.87 with individual item values ranging from 0.58 (Item 7) to 0.93 (Item 43). Bartlett’s test of sphericity was significant (χ²(990) = 7,093.55, *p* <.001), indicating that the data were suitable for EFA. Parallel analysis supported a seven-factor solution, as the first seven eigenvalues from the observed data exceeded those from randomly generated data. However, upon further examination, items from the original Muscle Building subscale (items: 7, 15, 29, 32, 35) did not load onto any of the seven factors. Given the theoretical salience of this subconstruct among women [49], we elected to retain an eight-factor model for subsequent CFA to ensure comparability with the sample of cisgender gay men in this investigation as well as other groups in the broader literature. The unrotated eight-factor solution accounted for 53.6% of the total variance. After oblique rotation, the eight-factor solution accounted for 16.22%, 13.61%, 11.60%, 8.12%, 7.77%, 8.27%, 5.64%, and 3.99% of the total variance, respectively, based on the structure matrix. Factor loadings from the pattern matrix ranged between 0.26 and 0.86 across the eight factors (Table 3). Six of the 45 items exhibited cross-loadings on multiple factors.

### Confirmatory factor analysis among cisgender lesbian women

We assessed multivariate normality using Mardia’s test, which indicated significant deviations from normality (*p* <.001). An eight-factor CFA model was conducted using the second split-half subsample of cisgender lesbian women (*n* = 286). The model demonstrated a good fit to the data (χ^2^(917) = 1208.40, CFI = 0.94, TLI = 0.93, and RMSEA = 0.05 (90% CI = 0.04, 0.06), SRMR = 0.07 (see Table 4). Each item significantly loaded onto its corresponding latent factor (loadings > 0.30, *p* <.001), except for Item 15 (“I thought about taking steroids as a way to get more muscular”), which had a non-significant loading (*p* =.08) on the Muscle Building factor. However, this item was retained in subsequent analyses to ensure a reliable test of configural invariance.

### Measurement invariance testing

To evaluate measurement invariance of the 45-item, 8-factor EPSI scale between cisgender gay men and cisgender lesbian women, a MG-CFA was conducted. We assessed configural, metric, scalar, and strict invariance following established thresholds for detecting significant differences in model fit [46, 50]. Fit indices for all models are presented in Table 5. The configural model demonstrated good fit (CFI = 0.968, TLI = 0.965, RMSEA = 0.039, SRMR = 0.061), indicating that the number of latent factors and the pattern of item loadings were similar across cisgender gay men and cisgender lesbian women. Next, we tested metric invariance to examine whether factor loadings are equivalent across groups. Although the ΔCFI was slightly beyond the acceptable threshold (ΔCFI = -0.012), the changes in RMSEA and SRMR were well within acceptable limits (ΔRMSEA = 0.006, ΔSRMR = 0.005), supporting the argument that the model’s loadings were close enough to be considered equivalent between groups (CFI = 0.956, TLI = 0.954, RMSEA = 0.045, SRMR = 0.066). Based on these results, we concluded that metric invariance was sufficiently supported.

**Table 4:** Confirmatory factor analysis fit indices for the eating pathology symptoms inventory (EPSI) across cisgender gay men and cisgender lesbian women

Group	N	χ²	df	CFI	RMSEA (90% CI)	SRMR
Cisgender Gay Men	462	1545.34	917	0.96	0.04 (0.04 − 0.05)	0.06
Cisgender Lesbian Women	286	1208.4	917	0.94	0.05 (0.04 − 0.06)	0.07

Weighted least squares with mean and variance adjusted (WLSMV) estimation method was used for each model

**Table 5:** Fit indices for measurement invariance of the EPSI across cisgender gay men and cisgender lesbian women

Model	RMSEA	ΔRMSEA	CFI	ΔCFI	SRMR	ΔSRMR
Configural	0.039	--	0.968	--	0.061	--
Metric	0.045	0.006	0.956	-0.012	0.066	0.005
Scalar	0.047	0.002	0.953	-0.003	0.067	0.001
Strict	0.047	0.001	0.952	-0.002	0.074	0.007

Δ = Change from the previous model. Differences in fit indices (Δ) are calculated relative to the preceding model

We then tested scalar invariance to examine the equality of item intercepts in addition to factor loadings. The fit indices showed small changes relative to the metric model (ΔCFI = -0.003, ΔRMSEA = 0.002, ΔSRMR = 0.001) with all indices remaining within acceptable thresholds (CFI = 0.953, TLI = 0.951, RMSEA = 0.047, SRMR = 0.067). Therefore, the factor loadings and item intercepts were stable across groups. Finally, the strict invariance model fit well (CFI = 0.950, TLI = 0.950, RMSEA = 0.047, SRMR = 0.074) with minimal differences from the scalar model (ΔCFI = -0.002, ΔRMSEA = 0.001, ΔSRMR = 0.007). This suggests that residual variances were consistent across cisgender gay men and cisgender lesbian women. Overall, these results provide support for the conclusion that the EPSI scale scores demonstrates configural, metric, scalar, and strict invariance between cisgender gay men and cisgender lesbian women. Although the ΔCFI for metric invariance slightly exceeded the recommended threshold, previous research suggested that ΔCFI can be sensitive in heterogeneous groups [51], and values between − 0.01 and − 0.02 should be interpreted cautiously rather than as definitive evidence of non-invariance [52]. Given that RMSEA and SRMR remained within acceptable limits, our approach aligned with recommendations to evaluate multiple indices together [46].

## Discussion

In this study, we examined the factor structure, reliability, and measurement invariance of the EPSI in samples of cisgender gay men and cisgender lesbian women. Overall, our findings largely support the utility of the original eight-factor model proposed by Forbush et al. (2013) [25], and the scale scores demonstrated adequate internal consistency within both groups. Although the eight-factor model generally provided a good fit across both groups, there were slight differences in fit quality between cisgender gay men and cisgender lesbian women. Moreover, the measure exhibited configural, metric, scalar, and strict invariance across cisgender gay men and cisgender lesbian women, indicating that meaningful comparisons can be made across these two groups. These findings align with those from previous EPSI validation studies [29].

The results yielded insights into the EPSI that warrant additional discussion Although the zero-order correlations among EPSI scales were generally modest (Table 2), the factor correlations derived from CFA were higher, as expected given that latent variables capture shared variance while reducing measurement error (Supplemental Table 2). Second, item 15 (“I thought about taking steroids as a way to get more muscular”) showed a notably lower factor loading on the Muscle Building scale among cisgender lesbian women. This may reflect differing gender-specific muscle-building behaviors between cisgender gay men and cisgender lesbian women [53–55]. This aligns with the Objectification Theory framework, which posits that societal pressures on women emphasize thinness and appearance more than muscularity (particularly muscle bulk; see Cunningham et al., 2022 [56] and Rodgers et al., 2018 [49]). Cisgender women may be less likely to internalize ideals related to muscle size than cisgender men, which could make especially extreme behaviors (e.g., steroid use) less relevant in the context of muscle-building in this population. In contrast, muscle dysmorphia and the pursuit of muscularity are more prevalent among men, particularly cisgender gay men [57], due to societal ideals that emphasize a muscular physique [58]. As such, steroid use may more closely align with the broader muscle-building construct.

### Strengths and limitations

A first limitation of this study is that we did not examine the intersection of various demographic groups. The demographic composition of our sample was predominantly White (84.8%), highly educated (79.7% holding a college degree or higher), and based in the U.S. (or its territories) which limits the generalizability of our findings. Additionally, we did not account for geographic location or urban/rural status, nor did we analyze potential age-related variations in eating disorder symptoms. Future research should address these gaps by incorporating psychometric and invariance testing across racial/ethnic, geographic, and age groups to improve the representativeness of findings. Second, mean scores on the EPSI scales were generally lower than those reported in prior studies of Richson et al., (2021) [29]. This finding may be due in part to the average age of participants in this investigation (M = 50.78 years); although eating disorders in later life is an understudied area [59], especially within sexual minority populations [2], the lower mean scores in this study may reflect the different demographic characteristics of our sample compared to those in previous studies [60]. Although some scales (e.g., Body Dissatisfaction at 0.95) showed very high internal consistency—often considered a sign of item redundancy [47] —they remained consistent with prior EPSI studies [25, 30]. As past research suggests, high reliability does not inherently indicate redundancy when inter-item correlations remain within acceptable limits [61]. The confirmatory factor analyses and invariance testing in this study were conducted with an eight-factor model, consistent with previous EPSI validation; however, future studies might consider whether alternative factor structures, such as a seven-factor model, better capture symptom nuances specific to cisgender lesbian women. Although this study established measurement invariance between cisgender gay men and cisgender lesbian women, we did not include other sexual and gender minority groups—such as bisexual, asexual, and non-binary individuals—in the current analysis. Given unique eating disorder considerations for specific sexual and gender minority subgroups, the factor structure of the EPSI should be examined in these distinct subgroups in future research.

## Conclusion

Our findings provide preliminary support for the usability of the eight-factor structure of the EPSI and suggest measurement invariance among cisgender gay men and cisgender lesbian women, indicating that the instrument may reliably assess diverse eating disorder symptoms in both populations. This allows clinicians and researchers to make meaningful comparisons across both groups and reliably characterize symptom severity within the cisgender gay men and lesbian women communities.

## Electronic supplementary material

Below is the link to the electronic supplementary material.

Supplementary Material 1

Supplementary Material 2

## Data Availability

Data from The PRIDE Study may be accessed through an Ancillary Study application (details at pridestudy.org/collaborate).

## Abbreviations

CFI: Comparative fit index
EPSI: Eating pathology symptoms inventory
IRB: Institutional review board
MG-CFA: Multi-group confirmatory factor analysis
PRIDE: Population research in identity and disparities for equality study
RMSEA: Root mean square error of approximation
SRMR: Standardized root mean square residual
TLI: Tucker-Lewis index

## References

[1] Nagata JM, Ganson KT, Austin SB. Emerging trends in eating disorders among sexual and gender minorities. Curr Opin Psychiatry. 2020;33:562–7. 32858597 10.1097/YCO.0000000000000645 PMC8060208

[2] Calzo JP, Blashill AJ, Brown TA, Argenal RL. Eating disorders and disordered weight and shape control behaviors in sexual minority populations. Curr Psychiatry Rep. 2017;19:49. 28660475 10.1007/s11920-017-0801-y PMC5555626

[3] Kamody RC, Grilo CM, Udo T. Disparities in DSM-5 defined eating disorders by sexual orientation among U.S. Adults. Int J Eat Disord. 2020;53:278–87. 31670848 10.1002/eat.23193

[4] Mason TB, Lewis RJ, Heron KE. Disordered eating and body image concerns among sexual minority women: A systematic review and testable model. Psychol Sex Orientat Gend Divers. 2018;5:397–422.

[5] Murray SB, Nagata JM, Griffiths S, Calzo JP, Brown TA, Mitchison D, et al. The enigma of male eating disorders: A critical review and synthesis. Clin Psychol Rev. 2017;57:1–11. 28800416 10.1016/j.cpr.2017.08.001

[6] Parker LL, Harriger JA. Eating disorders and disordered eating behaviors in the LGBT population: a review of the literature. J Eat Disord. 2020;8:51. 33088566 10.1186/s40337-020-00327-y PMC7566158

[7] Miskovic-Wheatley J, Bryant E, Ong SH, Vatter S, Le A, National Eating Disorder Research Consortium. Eating disorder outcomes: findings from a rapid review of over a decade of research. J Eat Disord. 2023;11:85. 37254202 10.1186/s40337-023-00801-3 PMC10228434

[8] da Luz FQ, Mohsin M, Jana TA, Marinho LS, Santos ED, Lobo I, et al. An examination of the relationships between eating-Disorder symptoms, difficulties with emotion regulation, and mental health in people with binge eating disorder. Behav Sci (Basel). 2023;13:234. 36975259 10.3390/bs13030234 PMC10045385

[9] Tan EJ, Raut T, Le LK-D, Hay P, Ananthapavan J, Lee YY, et al. The association between eating disorders and mental health: an umbrella review. J Eat Disord. 2023;11:51. 36973817 10.1186/s40337-022-00725-4 PMC10044389

[10] Devoe DJ, Dimitropoulos G, Anderson A, Bahji A, Flanagan J, Soumbasis A, et al. The prevalence of substance use disorders and substance use in anorexia nervosa: a systematic review and meta-analysis. J Eat Disord. 2021;9:161. 34895358 10.1186/s40337-021-00516-3 PMC8666057

[11] Bahji A, Mazhar MN, Hudson CC, Nadkarni P, MacNeil BA, Hawken E. Prevalence of substance use disorder comorbidity among individuals with eating disorders: A systematic review and meta-analysis. Psychiatry Res. 2019;273:58–66. 30640052 10.1016/j.psychres.2019.01.007

[12] Eskander N, Chakrapani S, Ghani MR. The risk of substance use among adolescents and adults with eating disorders. Cureus. 12:e10309. 10.7759/cureus.10309 PMC7544549 33052271

[13] Joiner TE, Robison M, McClanahan S, Riddle M, Manwaring J, Rienecke RD, et al. Eating disorder behaviors as predictors of suicidal ideation among people with an eating disorder. Int J Eat Disord. 2022;55:1352–60. 35792367 10.1002/eat.23770

[14] Convertino AD, Helm JL, Pennesi JL, Gonzales M, Blashill AJ. Integrating minority stress theory and the tripartite influence model: A model of eating disordered behavior in sexual minority young adults. Appetite. 2021;163:105204. 33741450 10.1016/j.appet.2021.105204

[15] Santoniccolo F, Rollè L. The role of minority stress in disordered eating: a systematic review of the literature. Eat Weight Disord. 2024;29:41. 38850334 10.1007/s40519-024-01671-7 PMC11162380

[16] Meyer IH. Prejudice, social stress, and mental health in lesbian, gay, and bisexual populations: conceptual issues and research evidence. Psychol Bull. 2003;129:674–97. 12956539 10.1037/0033-2909.129.5.674 PMC2072932

[17] Penwell TE, Bedard SP, Eyre R, Levinson CA. Eating disorder treatment access in the united States: perceived inequities among treatment seekers. Psychiatr Serv. 2024;75:944–52. 38716514 10.1176/appi.ps.20230193

[18] Putnick DL, Bornstein MH. Measurement invariance conventions and reporting: the state of the Art and future directions for psychological research. Dev Rev. 2016;41:71–90. 27942093 10.1016/j.dr.2016.06.004 PMC5145197

[19] Culbert KM, Sisk CL, Klump KL. A narrative review of sex differences in eating disorders: is there a biological basis? Clin Ther. 2021;43:95–111. 33375999 10.1016/j.clinthera.2020.12.003 PMC7902379

[20] Austen E, Griffiths S. Why do men stigmatize individuals with eating disorders more than women? Experimental evidence that sex differences in conformity to gender norms, not biological sex, drive eating disorders’ stigmatization. Eat Disord. 2019;27:267–90. 30052168 10.1080/10640266.2018.1499337

[21] Wiseman MC, Moradi B. Body image and eating disorder symptoms in sexual minority men: A test and extension of objectification theory. J Couns Psychol. 2010;57:154–66. 21133567 10.1037/a0018937

[22] Meneguzzo P, Collantoni E, Gallicchio D, Busetto P, Solmi M, Santonastaso P, et al. Eating disorders symptoms in sexual minority women: a systematic review. Eur Eat Disord Rev. 2018;26:275–92. 29708623 10.1002/erv.2601

[23] Fredrickson BL, Roberts T-A. Objectification theory: toward Understanding women’s lived experiences and mental health risks. Psychol Women Q. 1997;21:173–206.

[24] Watson LB, Grotewiel M, Farrell M, Marshik J, Schneider M. Experiences of sexual objectification, minority stress, and disordered eating among sexual minority women. Psychol Women Q. 2015;39:458–70.

[25] Forbush KT, Wildes JE, Pollack LO, Dunbar D, Luo J, Patterson K, et al. Development and validation of the eating pathology symptoms inventory (EPSI). Psychol Assess. 2013;25:859–78. 23815116 10.1037/a0032639

[26] Birgegård A, Isomaa R, Monell E, Bjureberg J. Validation of the eating pathology symptoms inventory (EPSI) in Swedish adolescents. J Eat Disord. 2024;12:68. 38802891 10.1186/s40337-024-01027-7 PMC11129359

[27] Sahlan RN, Blomquist KK, Bodell LP. Psychometric properties of the Farsi version of the eating pathology symptoms inventory (F-EPSI) among Iranian university men and women. J Eat Disord. 2022;10:67. 35534863 10.1186/s40337-022-00587-w PMC9082464

[28] Tang X, Forbush KT, Lui PP. Development and validation of the Chinese-language version of the eating pathology symptoms inventory. Int J Eat Disord. 2015;48:1016–23. 26171958 10.1002/eat.22423

[29] Richson BN, Forbush KT, Chapa DAN, Gould SR, Perko VL, Johnson SN, et al. Measurement invariance of the eating pathology symptoms inventory (EPSI) in adolescents and adults. Eat Behav. 2021;42:101538. 34247036 10.1016/j.eatbeh.2021.101538 PMC8518978

[30] Forbush KT, Wildes JE, Hunt TK. Gender norms, psychometric properties, and validity for the eating pathology symptoms inventory. Int J Eat Disord. 2014;47:85–91. 23996154 10.1002/eat.22180

[31] Perko VL, Forbush KT, Christensen KA, Richson BN, Chapa DAN, Bohrer BK, et al. Validation of the factor structure of the eating pathology symptoms inventory in an international sample of sexual minority men. Eat Behav. 2021;42:101511. 34004456 10.1016/j.eatbeh.2021.101511 PMC10042082

[32] Widaman K, Olivera-Aguilar M. Investigating measurement invariance using confirmatory factor analysis. In: Hoyle R, editor. Handbook of structural equation modeling. 2nd ed. Guilford; 2023. pp. 367–84.

[33] Lunn MR, Capriotti MR, Flentje A, Bibbins-Domingo K, Pletcher MJ, Triano AJ, et al. Using mobile technology to engage sexual and gender minorities in clinical research. PLoS ONE. 2019;14:e0216282. 31048870 10.1371/journal.pone.0216282 PMC6497300

[34] Obedin-Maliver J, Hunt C, Flentje A, Armea-Warren C, Bahati M, Lubensky ME et al. Engaging sexual and gender minority (SGM) communities for health research: Building and sustaining PRIDEnet. Journal of Community Engagement and Scholarship. 2024 [cited 2024 Jul 25];16. Available from: https://jces.ua.edu/articles/10.54656/jces.v16i2.484 10.54656/jces.v16i2.484 PMC11326444 39149568

[35] Morgan JF, Reid F, Lacey JH. The SCOFF questionnaire: a new screening tool for eating disorders. West J Med. 2000;172:164. 18751246 10.1136/ewjm.172.3.164 PMC1070794

[36] Stice E, Telch CF, Rizvi SL. Development and validation of the eating disorder diagnostic scale: a brief self-report measure of anorexia, bulimia, and binge-eating disorder. Psychol Assess. 2000;12:123–31. 10887758 10.1037//1040-3590.12.2.123

[37] Murray SB, Brown TA, Blashill AJ, Compte EJ, Lavender JM, Mitchison D et al. The development and validation of the muscularity-oriented eating test: A novel measure of muscularity-oriented disordered eating. Int J Eat Disord. 2019;52. 10.1002/eat.23144 31343090

[38] Forbush KT. Eating Pathology Symptoms Inventory (EPSI). In: Wade T, editor. Encyclopedia of Feeding and Eating Disorders. Singapore: Springer; 2016 [cited 2024 Nov 25]. pp. 1–3. Available from: 10.1007/978-981-287-087-2_50-1

[39] Revelle W. psych: Procedures for Personality and Psychological Research. Evanston, IL; 2018.

[40] Korkmaz S, Goksuluk D, Zararsiz G. MVN: an R package for assessing multivariate normality. R J. 2014;6:151–62.

[41] Rosseel Y, Lavaan. An R package for structural equation modeling. J Stat Softw. 2012;48:1–36.

[42] Hu L, Bentler PM. Cutoff criteria for fit indexes in covariance structure analysis: conventional criteria versus new alternatives. Struct Equation Modeling: Multidisciplinary J. 1999;6:1–55.

[43] Alwin D, Jackson D. Applications of simultaneous factor analysis to issues of factorial invariance. In: Jackson D, Borgatta E, editors. Factor analysis and measurement in sociological research: A multi-dimensional perspective. Beverly Hills: Sage; 1981. pp. 249–79.

[44] Van De Schoot R, Schmidt P, De Beuckelaer A, Lek K, Zondervan-Zwijnenburg M, Editorial. Measurement Invariance. Front Psychol. 2015 [cited 2024 Oct 11];6. Available from: https://www.frontiersin.org/journals/psychology/articles/10.3389/fpsyg.2015.01064/full 10.3389/fpsyg.2015.01064 PMC4516821 26283995

[45] Swami V, Barron D. Translation and validation of body image instruments: challenges, good practice guidelines, and reporting recommendations for test adaptation. Body Image. 2019;31:204–20. 30220631 10.1016/j.bodyim.2018.08.014

[46] Chen FF. Sensitivity of goodness of fit indexes to lack of measurement invariance. Struct Equ Model. 2007;14:464–504.

[47] Streiner DL. Starting at the beginning: an introduction to coefficient alpha and internal consistency. J Pers Assess. 2003;80:99–103. 12584072 10.1207/S15327752JPA8001_18

[48] Clark LA, Watson D. Constructing validity: basic issues in objective scale development. Psychol Assess. 1995;7:309–19.

[49] Rodgers RF, Franko DL, Lovering ME, Luk S, Pernal W, Matsumoto A. Development and validation of the female muscularity scale. Sex Roles. 2018;78:18–26.

[50] Cheung GW, Rensvold RB. Evaluating goodness-of-fit indexes for testing measurement invariance. Struct Equation Modeling: Multidisciplinary J. 2002;9:233–55.

[51] Rutkowski L, Svetina D. Assessing the hypothesis of measurement invariance in the context of large-scale international surveys. Educ Psychol Meas. 2014;74:31–57.

[52] Vandenberg RJ, Lance CE. A review and synthesis of the measurement invariance literature: suggestions, practices, and recommendations for organizational research. Organizational Res Methods. 2000;3:4–70.

[53] Feldman MB, Meyer IH. Eating disorders in diverse lesbian, gay, and bisexual populations. Int J Eat Disord. 2007;40:218–26. 17262818 10.1002/eat.20360 PMC2080655

[54] Piatkowski T, Whiteside B, Robertson J, Henning A, Lau EHY, Dunn M. What is the prevalence of anabolic-androgenic steroid use among women? A systematic review. Addiction. 2024;119:2088–100. 10.1111/add.16643 39134450

[55] Kutscher E, Arshed A, Greene RE, Kladney M. Exploring anabolic androgenic steroid use among cisgender gay, bisexual, and Queer men. JAMA Netw Open. 2024;7:e2411088. 38743422 10.1001/jamanetworkopen.2024.11088 PMC11094559

[56] Cunningham ML, Pinkus RT, Lavender JM, Rodgers RF, Mitchison D, Trompeter N, et al. The not-so-healthy appearance pursuit? Disentangling unique associations of female drive for toned muscularity with disordered eating and compulsive exercise. Body Image. 2022;42:276–86. 35841701 10.1016/j.bodyim.2022.06.002

[57] Convertino AD, Albright CA, Blashill AJ. Eating disorders and related symptomatology in sexual minority men and boys. In: Nagata JM, Brown TA, Murray SB, Lavender JM, editors. Eating Disorders in Boys and Men. Cham: Springer International Publishing; 2021 [cited 2024 Oct 11]. pp. 253–64. Available from: 10.1007/978-3-030-67127-3_17

[58] Murnen SK, Karazsia BT. A review of research on Men’s body image and drive for muscularity. The psychology of men and masculinities. Washington, DC, US: American Psychological Association; 2017. pp. 229–57.

[59] Mangweth-Matzek B, Kummer KK, Pope HG. Eating disorder symptoms in middle-aged and older men. Int J Eat Disord. 2016;49:953–7. 27173753 10.1002/eat.22550

[60] Rohde P, Stice E, Shaw H, Gau JM, Ohls OC. Age effects in eating disorder baseline risk factors and prevention intervention effects. Int J Eat Disord. 2017;50:1273–80. 28861902 10.1002/eat.22775 PMC5745064

[61] Clark LA, Watson D. Constructing validity: basic issues in objective scale development references. Psychol Assess. 1995;7:309–19.

