# Supplementary material for: Factor structure, internal consistency, and measurement invariance of the Eating Pathology Symptoms Inventory (EPSI) in a national U.S. sample of cisgender gay men and lesbian women
Source: J Eat Disord. 2025 May 14;13:83. doi: 10.1186/s40337-025-01277-z (PMC12076874; doi:10.1186/s40337-025-01277-z)
Supplement: Supplementary file 1 — Supplementary Material 1 [file 40337_2025_1277_MOESM1_ESM.docx]

| Table S1. CFA-derived factor correlations among the eight eating pathology symptoms inventory (EPSI) scales for cisgender gay men and cisgender lesbian women | | | | | | | | | |
| --- | --- | --- | --- | --- | --- | --- | --- | --- | --- |
|  |  | 1 | 2 | 3 | 4 | 5 | 6 | 7 | 8 |
| 1 | Body Dissatisfaction | — | .65 | .31 | .38 | .28 | .05 | .46 | .15 |
| 2 | Binge Eating | .65 | — | .09 | .43 | .21 | .06 | .42 | .16 |
| 3 | Cognitive Restraint | .51 | .34 | — | .10 | .24 | .53 | .42 | .44 |
| 4 | Purging | .37 | .48 | .22 | — | .30 | .06 | .26 | .20 |
| 5 | Restricting | .39 | .23 | .30 | .37 | — | .17 | .21 | .25 |
| 6 | Excessive Exercise | .23 | .23 | .61 | .21 | .23 | — | .20 | .68 |
| 7 | Negative Attitudes Toward Obesity | .49 | .48 | .45 | .32 | .18 | .38 | — | .12 |
| 8 | Muscle Building | .45 | .34 | .36 | .35 | .22 | .50 | .33 | — |
| Note. All p < .05. Lower correlations are cisgender gay men and upper correlations are cisgender lesbian women. | | | | | | | | | |
