# Supplementary material for: Factor structure, internal consistency, and measurement invariance of the Eating Pathology Symptoms Inventory (EPSI) in a national U.S. sample of cisgender gay men and lesbian women
Source: J Eat Disord. 2025 May 14;13:83. doi: 10.1186/s40337-025-01277-z (PMC12076874; doi:10.1186/s40337-025-01277-z)
Supplement: Supplementary file 2 — Supplementary Material 2 [file 40337_2025_1277_MOESM2_ESM.docx]

| Table S2. All exploratory factor analysis loadings (pattern matrix) in first split-half of cisgender gay men and cisgender lesbian women | | | | | | | | | | | | | | | | | | |
| --- | --- | --- | --- | --- | --- | --- | --- | --- | --- | --- | --- | --- | --- | --- | --- | --- | --- | --- |
|  |  | Cisgender Gay Men (*n* = 463) | | | | | | | |  | Cisgender Lesbian Women (*n* = 287) | | | | | | | |
|  |  | Factor Loadings | | | | | | | |  | Factor Loadings | | | | | | | |
| Item |  | 1 | 2 | 3 | 4 | 5 | 6 | 7 | 8 |  | 1 | 2 | 3 | 4 | 5 | 6 | 7 | 8 |
| *Binge Eating* |  |  |  |  |  |  |  |  |  |  |  |  |  |  |  |  |  |  |
| **3** |  | .61 | .13 | .04 | .02 | .06 | .00 | -.08 | -.04 |  | .59 | .20 | -.04 | .06 | .02 | .11 | -.07 | -.15 |
| **9** |  | .52 | .10 | .01 | -.02 | .01 | .01 | .03 | .04 |  | .57 | .16 | -.09 | -.01 | .07 | -.01 | .09 | .03 |
| **19** |  | .80 | .07 | -.04 | -.01 | .00 | -.01 | .05 | -.03 |  | .78 | .10 | -.05 | .01 | -.04 | .05 | .03 | -.05 |
| **28** |  | .66 | .09 | .03 | -.02 | .02 | .16 | .06 | .02 |  | .59 | .16 | -.11 | .00 | .17 | .09 | -.01 | .02 |
| **37** |  | .51 | .20 | -.04 | .12 | -.05 | .06 | -.03 | -.02 |  | .57 | .12 | .15 | .08 | .01 | .15 | -.07 | -.10 |
| **39** |  | .79 | -.10 | .03 | -.03 | -.01 | -.01 | .06 | .08 |  | .81 | -.05 | .07 | .04 | .00 | -.04 | .08 | .08 |
| **44** |  | .71 | .15 | -.01 | .05 | .00 | .04 | -.07 | -.01 |  | .67 | .24 | -.03 | .02 | .03 | .09 | -.07 | .48 |
| **45** |  | .74 | -.04 | .13 | .06 | .03 | -.01 | -.05 | -.04 |  | .75 | .10 | -.06 | -.06 | .04 | .11 | .08 | -.06 |
| *Body Dissatisfaction* | | |  |  |  |  |  |  |  |  |  |  |  |  |  |  |  |  |
| **1** |  | .17 | .68 | .13 | .06 | -.10 | -.03 | .07 | .28 |  | .01 | .84 | -.02 | .03 | .03 | .03 | .02 | .06 |
| **12** |  | .22 | .44 | .01 | .14 | .04 | .01 | .10 | .14 |  | .01 | .54 | -.02 | .12 | .07 | .05 | .21 | .09 |
| **18** |  | .11 | .74 | .05 | -.05 | .05 | .07 | .06 | -.02 |  | .12 | .75 | .04 | -.06 | .01 | .05 | .00 | .12 |
| **23** |  | .13 | .63 | -.04 | -.04 | -.02 | .09 | .05 | -.02 |  | .05 | .54 | .01 | .00 | -.03 | .16 | .06 | -.17 |
| **24** |  | .10 | .62 | .15 | -.09 | .07 | .05 | .00 | .05 |  | .11 | .77 | -.01 | -.05 | -.05 | -.04 | .04 | .00 |
| **25** |  | .09 | .73 | .12 | -.03 | .06 | .05 | .06 | -.01 |  | .06 | .83 | .07 | .02 | -.01 | -.02 | .01 | .10 |
| **34** |  | .01 | .62 | .13 | -.05 | .09 | .08 | -.04 | .10 |  | .04 | .78 | .03 | -.02 | -.04 | .02 | .07 | -.03 |
| *Cognitive Restraint* | | |  |  |  |  |  |  |  |  |  |  |  |  |  |  |  |  |
| **2** |  | -.16 | .09 | .48 | -.05 | -.07 | .11 | -.07 | .02 |  | -.09 | .10 | .56 | .22 | .05 | .18 | .12 | -.01 |
| **21** |  | -.06 | .12 | .75 | .07 | -.06 | -.10 | .05 | .36 |  | -.06 | .15 | .60 | .11 | .07 | .07 | .22 | .46 |
| **40** |  | -.15 | .15 | .48 | .02 | -.12 | .08 | .02 | -.03 |  | -.10 | .21 | .86 | .16 | .00 | .08 | .13 | .00 |
| *Excessive Exercise* | | |  |  |  |  |  |  |  |  |  |  |  |  |  |  |  |  |
| **5** |  | .00 | .15 | -.09 | .61 | .05 | .08 | .04 | .03 |  | -.02 | .11 | .13 | .61 | .03 | .05 | .08 | -.10 |
| **8** |  | .09 | -.05 | -.07 | .72 | .09 | .00 | .05 | .11 |  | -.02 | -.04 | -.08 | .84 | .02 | .03 | .04 | .05 |
| **22** |  | .06 | -.01 | -.03 | .28 | .08 | .01 | -.06 | -.08 |  | -.08 | .03 | .11 | .73 | -.06 | .04 | -.13 | -.07 |
| **31** |  | -.02 | -.12 | -.10 | .67 | .14 | .03 | .10 | -.03 |  | .01 | -.09 | .03 | .73 | .09 | -.04 | -.07 | -.07 |
| **41** |  | .08 | -.04 | -.02 | .61 | .08 | .05 | .07 | .08 |  | .18 | -.02 | -.04 | .65 | .01 | -.08 | .05 | .13 |
| *Muscle Building* | |  |  |  |  |  |  |  |  |  |  |  |  |  |  |  |  |  |
| **7** |  | .02 | -.09 | .03 | .13 | .75 | -.03 | .04 | -.05 |  | -.04 | -.05 | -.08 | .00 | .65 | .06 | .07 | .01 |
| **15** |  | .05 | .12 | .00 | -.06 | .66 | .06 | .00 | .00 |  | .06 | -.01 | .06 | -.01 | .37 | -.02 | -.07 | .09 |
| **29** |  | -.02 | .04 | .03 | .02 | .86 | .04 | .02 | .01 |  | .03 | -.02 | .00 | -.01 | .84 | -.03 | -.03 | -.04 |
| **32** |  | -.05 | .38 | -.03 | -.05 | .41 | .08 | -.05 | .16 |  | .12 | .30 | .06 | .10 | .31 | .03 | -.05 | .12 |
| **35** |  | .01 | -.07 | .01 | .22 | .54 | -.07 | -.05 | .06 |  | -.02 | -.06 | .11 | .06 | .51 | -.02 | .06 | .07 |
| *Negative Attitudes* | | |  |  |  |  |  |  |  |  |  |  |  |  |  |  |  |  |
| **14** |  | .05 | -.09 | .01 | .08 | -.03 | .76 | .05 | -.02 |  | .15 | -.09 | .05 | .03 | -.05 | .69 | .11 | .04 |
| **20** |  | .07 | -.09 | .03 | .41 | -.04 | .79 | .10 | -.03 |  | .13 | -.15 | -.08 | .05 | -.03 | .77 | .08 | .12 |
| **26** |  | -.04 | .09 | -.01 | .00 | -.03 | .74 | .00 | .10 |  | -.03 | .11 | -.03 | .01 | .03 | .75 | -.06 | .03 |
| **30** |  | .04 | .12 | .02 | .00 | .04 | .65 | -.07 | -.05 |  | .00 | .09 | .12 | -.04 | .09 | .68 | -.04 | -.08 |
| **38** |  | -.05 | .02 | .01 | -.05 | .07 | .86 | -.05 | .01 |  | -.07 | .05 | .02 | -.03 | -.01 | .83 | -.05 | -.03 |
| *Purging* |  |  |  |  |  |  |  |  |  |  |  |  |  |  |  |  |  |  |
| **11** |  | .09 | .10 | -.05 | -.01 | -.03 | .02 | .77 | -.01 |  | .07 | .15 | .05 | .00 | -.03 | -.07 | .57 | -.06 |
| **13** |  | -.05 | .02 | -.01 | .02 | .01 | .03 | .79 | .03 |  | -.04 | .02 | .08 | .00 | .05 | -.01 | .57 | .09 |
| **16** |  | .08 | -.12 | .22 | .02 | .25 | -.02 | .27 | .03 |  | .19 | -.02 | .07 | .16 | .17 | -.05 | .28 | .11 |
| **17** |  | -.01 | .03 | .17 | -.03 | .15 | -.07 | .53 | -.06 |  | -.02 | .03 | -.04 | .01 | .14 | .05 | .48 | -.04 |
| **27** |  | .28 | -.13 | .17 | -.02 | .09 | -.01 | .29 | .10 |  | .46 | -.28 | .41 | -.12 | -.03 | -.06 | .26 | .22 |
| **42** |  | -.08 | -.02 | -.02 | .05 | -.01 | .01 | .23 | .05 |  | .00 | -.03 | -.03 | -.10 | -.05 | .15 | .72 | -.14 |
| *Restricting* |  |  |  |  |  |  |  |  |  |  |  |  |  |  |  |  |  |  |
| **4** |  | -.04 | .00 | -.06 | -.06 | .10 | -.09 | .06 | .71 |  | .01 | .00 | -.09 | .05 | .04 | -.01 | .06 | .76 |
| **6** |  | -.08 | .11 | .02 | .06 | .01 | .00 | .03 | .73 |  | -.17 | .16 | .03 | -.05 | .01 | .13 | -.01 | .63 |
| **10** |  | .12 | -.06 | .02 | -.05 | -.05 | .09 | -.05 | .64 |  | -.08 | .04 | .04 | -.05 | -.02 | .04 | -.07 | .72 |
| **33** |  | -.02 | -.03 | .03 | .03 | -.09 | .02 | .01 | .83 |  | -.04 | .07 | .14 | -.06 | .03 | .03 | -.13 | .74 |
| **36** |  | .08 | -.09 | .02 | .04 | .10 | .03 | -.04 | .62 |  | .07 | .02 | -.02 | .08 | .01 | -.07 | .05 | .63 |
| **43** |  | .11 | .15 | .05 | -.02 | .00 | .05 | .41 | .74 |  | .15 | .09 | -.09 | -.03 | -.05 | .10 | .03 | .48 |
